# After-Hours Service Demands and Dentist Well-Being: Unpacking the Roles of Compassion Satisfaction and Organizational Support

**DOI:** 10.3390/healthcare14091239

**Published:** 2026-05-04

**Authors:** Fatma Mansour Abdulmawla, Sami Mohammad, Ayse Arslan

**Affiliations:** Department of Business Administration, Cyprus Health and Social Sciences University, 99700 Güzelyurt, Turkey; sami.hatem@kstu.edu.tr (S.M.);

**Keywords:** after-hours service demands, compassion satisfaction, perceived organizational support, overall work experience, JD-R theory, COR theory, dentists’ well-being

## Abstract

**Background/Objective:** This study examines how after-hours service demands (AHSD) are associated with dentists’ overall work experience (OWE) through the mediating role of compassion satisfaction (CS) and the moderating role of perceived organizational support (POS). Grounded in the Job Demands–Resources (JD-R) and Conservation of Resources (COR) theories, the study investigates how job demands, emotional resources, and organizational support jointly relate to dentists’ psychological well-being. **Methods:** Data were collected from 450 dentists across seven major Libyan cities—Tripoli, Benghazi, Misrata, Sabha, Al Bayda, Zawiya, and Derna—using a structured online questionnaire administered between May and August 2025. **Results:** Partial least squares structural equation modeling (PLS-SEM) results indicated that AHSD were positively associated with both CS and OWE, suggesting that demanding work conditions may, under certain conditions, coincide with more positive professional experiences when perceived as meaningful and supported. CS partially mediated the AHSD–OWE relationship, highlighting its role as a key emotional resource linked to more favorable work experiences. In addition, POS moderated the relationships between AHSD and CS, and between AHSD and OWE, although the effects were relatively modest, indicating that organizational support may provide incremental support in how dentists experience demanding work conditions rather than fundamentally altering these relationships. The moderated mediation analysis further suggested that the indirect association between AHSD and OWE via CS was stronger at higher levels of POS. **Conclusions:** Overall, the findings refine JD-R and COR perspectives by indicating that job demands, emotional resources, and organizational support are jointly associated with dentists’ work-related well-being in a high-demand healthcare context.

## 1. Introduction

Dental professionals face uniquely demanding work environments characterized by intensive technical precision, emotional labor, and sustained patient interaction. Beyond clinical duties, many dentists are also responsible for managing their own practices, balancing patient care with administrative and business responsibilities [1]. These dual pressures—clinical and managerial—can intensify psychological strain and reduce opportunities for recovery. In the Libyan healthcare context, these challenges are further intensified by structural constraints, including workforce shortages, uneven distribution of healthcare services, and rising patient demand, which collectively place substantial pressure on dental professionals [2,3]. As a result, dentists are frequently required to extend their working hours beyond standard schedules, particularly to address emergency cases and urgent patient needs, leading to prolonged work exposure and limited recovery time [4,5]. Such conditions create a high-demand work environment in which long working hours and after-hours responsibilities are not exceptional but routine, making it essential to examine how these demands shape dentists’ overall work experience and well-being. At the same time, these conditions reflect a resource-constrained and institutionally fragile setting; therefore, the patterns observed in this study should be interpreted in relation to the Libyan context rather than assumed to apply uniformly to more stable or high-resource healthcare systems.

After-hours service demands (AHSD) represent one of the most salient yet understudied stressors in dental practice. Dentists are often expected to remain available for emergencies, callbacks, or unscheduled treatments beyond regular working hours. These demands can intrude upon personal time, hinder recovery, and disrupt family and social life [6,7]. Research in related fields consistently links extended work availability to impaired recovery and increased stress [8,9]. However, existing well-being research has predominantly focused on nurses and physicians, while dentists remain comparatively underexplored despite their distinct workload structures and professional responsibilities [10,11]. This highlights a clear empirical gap in understanding how after-hours service demands influence dentists’ work-related outcomes.

Importantly, job demands are not exclusively detrimental. Providing urgent care may enhance professional meaning and fulfillment, reflecting the dual nature of job demands [12]. In this context, compassion satisfaction (CS)—defined as the sense of fulfillment derived from helping others—emerges as a critical psychological resource associated with engagement and resilience [13,14,15]. Similarly, perceived organizational support (POS) plays a key role in shaping how employees interpret demanding work conditions, with supportive environments enabling individuals to perceive demands as meaningful rather than burdensome [16,17,18].

This study draws on the Job Demands–Resources (JD-R) theory [19] and the Conservation of Resources (COR) theory [20] to conceptualize these dynamics. Within this framework, after-hours service demands function as job demands, compassion satisfaction as a personal resource, and perceived organizational support as an organizational resource influencing employees’ responses to demanding conditions [21,22]. Overall work experience (OWE) is conceptualized as a holistic indicator of occupational well-being, capturing both affective and cognitive evaluations of work, including satisfaction, engagement, and emotional connection [23,24,25].

Accordingly, this study aims to empirically examine these interrelated mechanisms within the Libyan healthcare context. By focusing on dentists—a professional group that remains underrepresented in occupational well-being research despite facing distinct and demanding work conditions—this study addresses a critical empirical and contextual gap. More specifically, the study makes three mechanism-level contributions. First, it conceptualizes after-hours service demands in dentistry as a dual-nature demand that may be associated not only with strain but also with professional meaning, thereby moving beyond JD-R applications that treat demanding work primarily as an impairment trigger. Second, it positions compassion satisfaction not merely as a positive correlate of well-being but as a psychological mechanism through which demanding work can be translated into a more favorable overall work experience. Third, it specifies perceived organizational support as a conditional organizational resource that shapes whether after-hours demands are interpreted as burdensome intrusions or meaningful professional contributions. These contributions are particularly relevant in dentistry because dentists often combine clinical responsibility, emergency responsiveness, professional autonomy, and administrative accountability to a greater extent than many other healthcare workers, making the demand–resource dynamic structurally distinct rather than simply comparable to nursing or physician settings. These insights also generate practical implications for healthcare administrators and policymakers seeking to enhance workforce resilience, engagement, and retention. To achieve these aims, the following research questions guide the investigation:

- RQ1: How do AHSD directly influence dentists’ OWE?
- RQ2: What role does CS play in mediating the relationship between AHSD and OWE?
- RQ3: To what extent does POS moderate the relationships between AHSD and CS, and between AHSD and OWE?
- RQ4: Does POS moderate the indirect effect of AHSD on OWE through CS?

Together, these research questions aim to uncover the interconnected pathways through which job demands, emotional resources, and organizational support interact to shape dentists’ professional fulfillment and well-being, within a context marked by structural pressure and limited organizational slack, offering both theoretical advancement within the JD-R and COR frameworks and practical guidance for promoting sustainable work engagement in oral healthcare settings.

## 2. Literature Review and Theoretical Background

### 2.1. Underpinning Theories: JD-R and COR

The Job Demands–Resources (JD-R) theory provides a structured framework for examining how job characteristics influence employee well-being and performance [19]. It distinguishes between job demands, which require sustained effort, and job resources, which support goal attainment and facilitate positive work outcomes [26,27,28]. Rather than providing a general explanation, this study applies JD-R specifically to classify the core constructs under investigation, where after-hours service demands (AHSD) represent job demands, compassion satisfaction (CS) reflects a personal resource, perceived organizational support (POS) represents an organizational resource, and overall work experience (OWE) captures the well-being outcome.

Within this framework, AHSD constitute a salient job demand in dental settings due to extended availability for emergency care and patient needs beyond regular working hours [29,30]. Consistent with the JD-R perspective, such demands are not assumed to be uniformly detrimental; instead, their effects depend on the availability of supporting resources [31]. POS functions as an organizational resource that facilitates more positive interpretations of demanding work conditions, while CS operates as a personal resource that enables individuals to derive meaning and emotional fulfillment from their professional activities [15,32,33,34]. Accordingly, JD-R is used in this study to explain how demands and resources are structurally related to work-related outcomes.

To complement this structural perspective, the Conservation of Resources (COR) theory is employed to explain the underlying mechanism through which resources are depleted and replenished over time [35]. The COR theory posits that individuals strive to retain and build valued resources, and that stress arises when these resources are threatened or lost [36]. In the context of this study, AHSD may contribute to resource depletion by reducing recovery opportunities, whereas POS (external resource) and CS (internal resource) support resource preservation and gain processes [16,37]. Thus, the COR theory is not used to redefine demands and resources, but to clarify how their dynamic interaction influences employees’ psychological states.

By combining JD-R and COR, this study distinguishes clearly between structure and mechanism; JD-R explains how AHSD, CS, POS, and OWE are conceptually related, while COR explains how resource loss and gain processes shape these relationships under demanding work conditions.

### 2.2. Employee Well-Being in the Healthcare Sector

Employee well-being in healthcare is a critical determinant of both workforce sustainability and service quality [38]. Healthcare professionals frequently operate under demanding conditions characterized by high responsibility and emotional intensity, increasing susceptibility to stress and fatigue [37]. Despite extensive research in this domain, prior studies have predominantly focused on physicians and nurses, with comparatively limited attention given to dentists, despite their distinct work conditions [7].

Dentistry presents a unique occupational context in which clinical responsibilities are often combined with administrative pressures and extended work demands [6]. These characteristics make dentists particularly sensitive to fluctuations in job demands and available resources, highlighting the need to examine well-being through an integrated theoretical lens.

In this study, OWE is conceptualized as a holistic indicator of well-being that captures individuals’ overall evaluative and emotional experiences at work [24]. Unlike job satisfaction or work engagement, OWE reflects a broader construct that integrates cognitive evaluation and affective experience, making it suitable for assessing well-being in complex professional environments [39,40].

Within the JD-R and COR frameworks, well-being is understood as an outcome shaped by the interaction between job demands and available resources. In this context, AHSD represent a key demand, while CS and POS function as personal and organizational resources that influence how these demands are experienced. This perspective enables a more focused examination of how work conditions translate into overall work experience among dentists.

### 2.3. After-Hours Demands and Overall Work Experience

AHSD represent a salient job demand in dentistry, as practitioners are frequently required to provide care beyond regular working hours, including emergency treatments and patient follow-ups [4,18,29]. Such demands require sustained cognitive and emotional effort and may limit opportunities for recovery, thereby influencing employees’ overall work experience.

Existing research suggests that extended work demands are associated with fatigue, impaired recovery, and reduced work–life balance [5,41]. From a job demands perspective, these conditions may negatively affect work-related evaluations by increasing strain and emotional exhaustion. However, within the JD-R framework, job demands are not inherently detrimental and may also function as challenge demands when associated with meaningful outcomes [19].

In dentistry, after-hours interventions often involve alleviating patient pain or restoring function, enabling practitioners to directly observe the impact of their work. This immediacy may contribute to perceptions of professional meaning and may be associated with more positive evaluations of work experience under certain conditions, despite the demanding nature of such tasks [42].

From a resource perspective, the net effect of AHSD depends on the balance between resource depletion and potential resource gain [20,43]. Accordingly, AHSD can be conceptualized as a dual-function demand that may either undermine or be positively associated with work experience depending on individual appraisal and the availability of supporting resources [44].

Importantly, these theoretical relationships should be interpreted within the specific context of the Libyan dental sector and do not imply universal or deterministic effects across all professional or institutional settings. Based on the JD-R and COR frameworks, AHSD may function as a challenge demand that is positively associated with overall work experience. Therefore, the following hypothesis is proposed:

**H1.**  *After-hours service demands are significantly associated with dentists’ overall work experience.*

### 2.4. After-Hours Demands and Compassion Satisfaction

AHSD require dentists to respond to patient needs beyond standard working hours, often under time pressure and emotional intensity [45]. Such demands are typically associated with increased strain and reduced recovery [5,41].

However, emerging evidence suggests that job demands may also generate positive psychological outcomes when linked to meaningful work experiences. Compassion satisfaction (CS), defined as the fulfillment derived from helping others, represents a key psychological resource in healthcare settings [15].

In dentistry, the immediate impact of treatment outcomes allows practitioners to directly observe the benefits of their work. Providing care during after-hours periods may strengthen perceptions of purpose and may be associated with increased professional fulfillment in certain contexts, rather than uniformly producing positive outcomes [42,46].

From a JD-R perspective, CS functions as an internal resource that enables individuals to reinterpret demanding situations more positively, transforming after-hours work into a meaningful experience [19]. Thus, AHSD may contribute to higher CS when these demands are perceived as meaningful and are supported by sufficient psychological and organizational resources [47].

These relationships are context-dependent and should be interpreted cautiously, particularly within the Libyan healthcare environment characterized by specific structural and resource constraints. Based on this reasoning, AHSD can stimulate compassion satisfaction when experienced as meaningful professional contributions. Therefore, the following hypothesis is proposed:

**H2.**  *After-hours service demands are positively associated with compassion satisfaction among dentists.*

### 2.5. Compassion Satisfaction and Overall Work Experience

Compassion satisfaction (CS) represents a positive psychological state reflecting fulfillment, meaning, and accomplishment derived from helping others [15]. As a personal resource, CS plays a central role in shaping how individuals evaluate their work experiences.

Empirical research consistently shows that CS is associated with higher engagement, job satisfaction, and reduced burnout [13,48]. These findings indicate that CS contributes directly to more favorable work-related evaluations and overall well-being.

In dentistry, the ability to alleviate patient pain and deliver visible outcomes reinforces a sense of professional purpose. This strengthens emotional attachment to work and enhances overall work experience.

From a resource-based perspective, CS replenishes emotional energy and supports resilience, enabling individuals to maintain positive work evaluations under demanding conditions [49,50]. As a result, dentists with higher CS are more likely to experience their work positively despite job demands.

Thus, AHSD may contribute to higher CS when these demands are perceived as meaningful and are supported by sufficient psychological and organizational resources. Accordingly, CS functions as a key determinant of overall work experience. Therefore, the following hypothesis is proposed:

**H3.**  *Compassion satisfaction is positively associated with dentists’ overall work experience.*

### 2.6. Mediating Role of Compassion Satisfaction

While after-hours service demands (AHSD) influence work-related outcomes, their effects are not necessarily direct. Compassion satisfaction (CS) is proposed as the mechanism through which AHSD shapes overall work experience (OWE).

AHSD introduce additional effort and time demands, which may increase strain. However, when these demands are associated with meaningful patient care, they may also generate emotional fulfillment. CS captures this positive psychological response [51].

Empirical evidence indicates that CS enhances engagement and reduces burnout [13,48]. Thus, individuals who experience higher CS are more likely to interpret demanding work as meaningful rather than burdensome.

From a resource perspective, CS represents an internal resource that converts demanding work conditions into positive work-related evaluations. In the absence of CS, AHSD are more likely to be perceived negatively, leading to reduced work experience [52,53,54].

This mediating mechanism reflects a conditional process rather than a universally consistent effect. Accordingly, CS explains how AHSD influence OWE by transforming demanding experiences into meaningful outcomes. Therefore, the following hypothesis is proposed:

**H4.**  *Compassion satisfaction mediates the relationship between after-hours service demands and dentists’ overall work experience.*

### 2.7. Moderating Role of Organizational Support

Perceived organizational support (POS) reflects the extent to which employees believe their organization values their contributions and cares about their well-being [34]. As an organizational resource, POS influences how job demands are interpreted and managed.

In dentistry, where professionals often operate in relatively independent settings, POS becomes a critical contextual factor shaping responses to after-hours demands [55].

POS is expected to moderate the relationship between AHSD and CS. When organizational support is high, after-hours demands are more likely to be perceived as valued contributions, enhancing compassion satisfaction. In contrast, low support may lead to negative interpretations of the same demands [56,57,58].

Similarly, POS is expected to moderate the relationship between AHSD and OWE. High support provides emotional and instrumental resources that help individuals sustain positive work evaluations under demanding conditions [18,59,60].

Furthermore, POS conditions the strength of the indirect relationship between AHSD and OWE through CS, as supportive environments reinforce the positive interpretation of demanding work experiences.

The moderating effects of organizational support should therefore be interpreted as context-sensitive rather than universally generalizable. Based on this reasoning, the following hypotheses are proposed:

**H5.**  *Perceived organizational support positively moderates the relationship between after-hours service demands and compassion satisfaction, such that the relationship is stronger when organizational support is high.*

**H6.**  *Perceived organizational support positively moderates the relationship between after-hours service demands and dentists’ overall work experience, such that the relationship is stronger when organizational support is high.*

**H7.**  *The indirect effect of after-hours service demands on overall work experience through compassion satisfaction is stronger at higher levels of perceived organizational support.*

## 3. Methodology

### 3.1. Research Context

Libya presents a complex and challenging environment for examining the well-being of dental professionals due to its prolonged political instability, economic decline, and weakened healthcare infrastructure since 2011. Once a model for healthcare provision in the region, the Libyan health system has deteriorated under the strain of continuous conflict and limited governance, resulting in unequal service delivery, workforce shortages, and declining care quality [2,61]. Within this fragile system, the dental sector faces additional pressures, including an uncontrolled surge in the number of dental schools and graduates, often at the expense of educational standards and professional preparedness [62]. The oversupply of dentists, coupled with limited employment opportunities and inadequate institutional support, has intensified competition and increased reliance on after-hours practice as a means of sustaining income. Moreover, oral health indicators reflect a growing public health burden, with high and rising prevalence of untreated dental caries attributed to limited preventive programs, reduced access to care, and low awareness of oral hygiene [63]. These systemic challenges have created a demanding work environment where dentists are frequently required to extend their working hours, leading to heightened physical and emotional strain. Against this backdrop, Libya offers a particularly relevant context for exploring how after-hours service demands influence dentists’ overall work experience and psychological well-being within a strained healthcare system.

### 3.2. Sample and Data Collection

This study employed a quantitative, cross-sectional research design to examine how after-hours service demands influence dentists’ work experience and psychological well-being in Libya. The sampling frame was drawn from dental-care institutions across the country, encompassing both public and private healthcare facilities that operate under different administrative authorities [2]. According to the Libyan Ministry of Health, the national health system includes 96 hospitals, 25 specialized departments, 1355 main medical centers, 37 clinics, and 17 quarantine departments [64]. Because an exact national registry of practicing dentists was not publicly available, the broader official healthcare workforce figure reported by the World Health Organization (WHO), which lists 21,568 registered doctors in Libya, was used only as an approximate population reference for sample size estimation rather than as the actual target population of the study. Based on Taro Yamane’s [65] formula, with a 95% confidence level and a 5% margin of error, a minimum sample size of 393 was deemed adequate. This threshold was exceeded, as the final sample comprised 450 valid responses, thereby increasing statistical adequacy. Furthermore, the sample size satisfies the minimum requirements for Partial Least Squares Structural Equation Modeling (PLS-SEM) analysis, which recommends a sample size exceeding ten times the maximum number of structural paths directed at any construct [66].

However, due to the absence of a comprehensive national registry of practicing dentists and the ongoing socio-political instability in Libya, probability sampling techniques were not feasible. Therefore, a convenience (nonprobability) sampling approach was adopted. Participants were recruited based on the following inclusion criteria: (1) being a licensed practicing dentist in Libya, and (2) currently employed in either public or private dental-care institutions. Dentists were recruited from public hospitals, medical polyclinics, specialized dental centers, and private dental practices across major Libyan cities—Tripoli, Benghazi, Misrata, Sabha, Al Bayda, Zawiya, and Derna—to ensure broad geographic and institutional coverage [67]. While this approach enabled access to a diverse sample, it may also introduce sampling bias due to differential participation across regions and recruitment channels. In particular, dentists affiliated with active professional networks or urban centers may be overrepresented, whereas those in less-connected or rural areas may be underrepresented. The use of convenience sampling is justified in contexts where population frames are incomplete and access to respondents is constrained; however, it may introduce selection bias and limit the generalizability of the findings [68].

Data were collected between May and August 2025 using a self-administered, online-based questionnaire distributed through Google Forms. The survey link was disseminated via multiple recruitment channels, including professional dental associations, institutional mailing lists, and targeted social-media groups of practicing dentists, thereby maximizing reach and participation. The survey introduction clearly outlined the study’s purpose and assured respondents of anonymity, confidentiality, and voluntary participation. Prior to full deployment, the questionnaire was pilot-tested with 25 dentists from different regions to ensure clarity, relevance, and cultural appropriateness, and minor revisions were implemented accordingly [69].

A total of 1117 survey invitations were distributed, yielding 450 completed and usable responses, corresponding to a response rate of 40.3%, which is considered acceptable in healthcare survey research [70]. Two follow-up reminders were sent at one-week intervals to improve participation rates. All returned questionnaires were systematically screened for completeness and consistency. Responses with missing data or incomplete answers were excluded from the analysis. As a result, the final dataset contained no missing values, ensuring data integrity for subsequent statistical analysis.

The final sample included dentists from both public and private sectors, with varied professional experience and demographic characteristics, reflecting the heterogeneous nature of Libya’s dental workforce. Although efforts were made to ensure geographic diversity, such diversity does not guarantee full representativeness of the national dental workforce. Therefore, the findings should be interpreted with caution in terms of external validity, as the sample may not fully capture the distribution and characteristics of all practicing dentists across Libya. The data were analyzed using PLS-SEM to test the hypothesized direct, mediating, and moderating relationships.

Table 1 summarizes the demographic profile of the respondents. The sample demonstrates diversity in gender, age, education, experience, and geographic distribution. Most respondents were aged between 30 and 39 years (41.6%), held a Bachelor’s degree (61.8%), and worked full-time (85%). Approximately 39% had 5–10 years of experience, and respondents were distributed across major Libyan cities, ensuring broad but not fully representative coverage of the national dental workforce.

### 3.3. Measures

The survey instrument was developed by adapting well-established and previously validated measurement scales to ensure construct validity and theoretical alignment with the study objectives. All measurement instruments have been widely validated in prior healthcare and organizational research contexts, supporting their applicability and robustness. Since the research was conducted in an Arabic-speaking environment, the original English items were translated and back-translated following Brislin’s [71] procedure to ensure linguistic accuracy and conceptual equivalence. In addition to translation, minor contextual adaptations were made to align the wording with dental practice settings in Libya while preserving the original meaning of the constructs.

To ensure content and face validity, two pretests were conducted with subject-matter experts in oral health and organizational behavior, who evaluated the clarity, relevance, and cultural appropriateness of the items. A pilot study involving 25 dentists from different regions of Libya was subsequently conducted to assess item comprehension and contextual suitability. These respondents were excluded from the final sample. Minor refinements were made based on their feedback, thereby enhancing clarity and reliability. The final questionnaire employed a five-point Likert scale ranging from 1 (“strongly disagree”) to 5 (“strongly agree”), ensuring consistency and ease of response.

Four constructs were measured using established multi-item scales (see Appendix A, Table A1). After-hours service demands (AHSD) were assessed using a five-item scale adapted from Bakker and Demerouti [72] and Xanthopoulou et al. [73], capturing the extent to which dentists are required to work beyond regular hours (e.g., “I am frequently required to provide dental care beyond regular working hours”). Compassion satisfaction (CS) was measured using the ten-item Professional Quality of Life Scale (ProQOL V) developed by Stamm [74], reflecting the fulfillment derived from helping others (e.g., “I get satisfaction from being able to help people”). Perceived organizational support (POS) was evaluated using the eight-item short version of the Survey of Perceived Organizational Support by Eisenberger et al. [34], capturing employees’ perceptions of organizational care and recognition (e.g., “My organization really cares about my well-being”). Overall work experience (OWE) was measured using eight items adapted from Zheng et al. [75] and Schaufeli et al. [76], reflecting satisfaction, engagement, and emotional connection to work (e.g., “Overall, I am satisfied with my work experience”).

All constructs demonstrated satisfactory internal consistency reliability. Cronbach’s alpha values were 0.824 for AHSD, 0.879 for CS, 0.836 for POS, and 0.813 for OWE, exceeding the recommended threshold of 0.70. These results confirm the reliability and stability of the measurement scales within the Libyan dental context. Overall, the combination of validated scales, rigorous translation procedures, pilot testing, and reliability assessment ensures the robustness and appropriateness of the measurement model.

### 3.4. Common Method Variance

Because this study relied on self-reported survey data, the possibility of common method variance (CMV)—systematic error arising from the measurement method rather than the constructs being examined—was carefully addressed to support the validity of the findings [77]. Several procedural remedies were applied during the design and administration of the questionnaire: respondents were guaranteed anonymity and confidentiality, participation was voluntary, and items were adapted from well-validated scales and refined through pilot testing to minimize ambiguity and social desirability bias [78]. These procedures were intended to reduce evaluation apprehension and lower the likelihood of artificially inflated associations among variables. To statistically assess CMV, Harman’s single-factor test was conducted, revealing that the largest single factor accounted for 34.76% of the total variance, which is below the 50% threshold commonly considered problematic [77]. Recognizing the limitations of this approach, the full collinearity test recommended by Kock [79] was also applied; the variance inflation factor (VIF) values for all constructs ranged between 1.109 and 1.949, remaining well below the cut-off value of 3.3 [80]. Taken together, these results suggest that CMV is unlikely to constitute a severe threat to the validity of the findings.

Nevertheless, these procedural and statistical checks reduce rather than eliminate the possibility of residual common method bias. In a cross-sectional design where all variables were collected from the same respondents at a single point in time, single-source self-reports may inflate path estimates, and the pattern of fully significant hypothesized relationships may partly reflect shared method variance or positively aligned response tendencies rather than the true strength of the associations alone. Therefore, although the available evidence indicates that CMV was not a dominant problem in this study, the reported associations should be interpreted with this limitation in mind, and future research is encouraged to employ multi-source data collection, time-lagged designs, or objective indicators to strengthen causal and discriminant validity.

### 3.5. Analysis

Data analysis was conducted using Partial Least Squares Structural Equation Modeling (PLS-SEM), a variance-based technique suitable for examining complex models incorporating mediation and moderation effects. PLS-SEM was selected due to its robustness in handling non-normal data, model complexity, and predictive-oriented research objectives [66,81]. The analysis followed the two-step approach recommended by Hair et al. [80], involving (1) assessment of the measurement model (reliability and validity) and (2) evaluation of the structural model (hypothesis testing). The analysis was performed using SmartPLS 4.1.0.9, with bootstrapping (5000 resamples) applied to assess the significance of path coefficients and indirect effects [82]. SPSS version 25.0 (IBM Corp, Armonk, NY, USA) was used for descriptive statistics.

**Figure 1:**
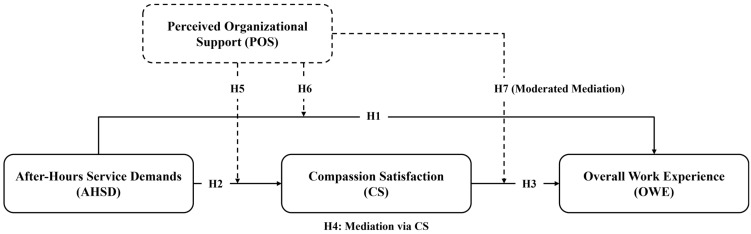
Conceptual Model. Note: Solid arrows represent direct effects, while dashed arrows represent moderating and moderated mediation effects.

The conceptual model, comprising direct (H1–H3), mediating (H4), moderating (H5–H6), and conditional indirect relationships (H7), was tested using PLS-SEM. Specifically, AHSD was modeled as the independent variable, OWE as the dependent variable, CS as a mediator, and POS as a moderator. This specification enables the simultaneous estimation of direct, indirect, and interaction effects within a unified analytical framework. Figure 1 presents the conceptual model with all hypothesized relationships (H1–H7).

## 4. Results

### 4.1. Measurement Model Assessment

The measurement model was evaluated to assess internal consistency reliability, convergent validity, and discriminant validity in accordance with established PLS-SEM guidelines [66,80]. As shown in Table 2, all retained indicator loadings exceeded the recommended threshold of 0.70, with a few acceptable values above 0.60, confirming that the observed variables adequately represent their respective latent constructs [83]. Two items from the perceived organizational support scale (POS5 and POS7) were removed due to low outer loadings below the recommended threshold of 0.70, consistent with established indicator reliability criteria. Importantly, POS5 and POS7 were reverse-coded items, and their weak psychometric performance may reflect wording difficulty, reverse-coding sensitivity, or limited contextual fit within this sample. Their removal was therefore undertaken to preserve construct reliability and convergent validity rather than to artificially improve the model. The retained POS items continued to capture the core conceptual domain of perceived organizational support.

Internal consistency reliability was confirmed, with Cronbach’s alpha (CA) values ranging from 0.813 to 0.879 and composite reliability (CR) values ranging from 0.833 to 0.895, exceeding the recommended threshold of 0.70 [66,84]. Convergent validity was established as all constructs achieved average variance extracted (AVE) values greater than 0.50, indicating that each construct explains more than half of the variance of its indicators [85]. Descriptive statistics, including means and standard deviations, are reported in Table 2, and 95% confidence intervals were examined to ensure stability of the estimates.

To assess discriminant validity, both the Fornell–Larcker criterion and the Heterotrait–Monotrait (HTMT) ratio were employed. As presented in Table 3, the square root of each construct’s AVE exceeded its inter-construct correlations, satisfying the Fornell–Larcker criterion [85]. Additionally, all HTMT values were below the conservative threshold of 0.85, confirming that the constructs are empirically distinct [86].

Variance Inflation Factor (VIF) values were also examined and found to be below the recommended threshold of 3.3, indicating the absence of multicollinearity and providing additional evidence that common method bias is not a serious concern [79,80].

Collectively, these results confirm that the measurement model demonstrates satisfactory reliability, convergent validity, and discriminant validity, supporting its suitability for subsequent structural model analysis.

### 4.2. Structural Model Assessment: Direct and Indirect Effects

The structural model was assessed to evaluate the hypothesized relationships among the latent constructs and to determine the model’s explanatory power and predictive relevance [87]. Prior to hypothesis testing, collinearity diagnostics confirmed that all variance inflation factor (VIF) values were below 3.3, indicating no multicollinearity concerns [79]. In line with PLS-SEM methodological guidelines, model fit indices such as Chi-square and NFI were not considered, and the evaluation focused on variance explained (R^2^), predictive relevance (Q^2^), and effect sizes (f^2^).

**Figure 2:**
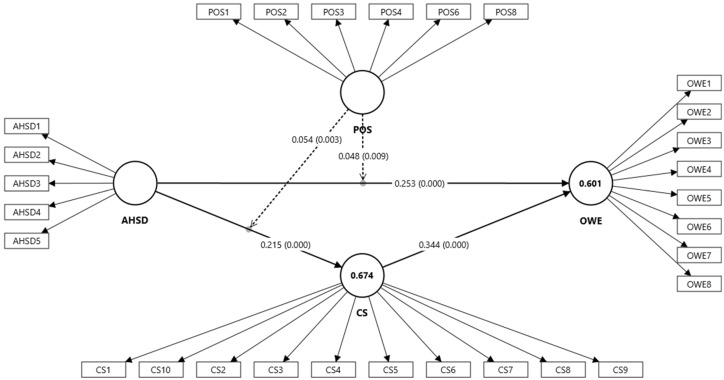
Path analysis. Note: Circles represent latent constructs; solid arrows indicate direct effects, and dashed arrows indicate moderating effects.

As shown in Figure 2, the R^2^ values indicate that the model explains 67.4% of the variance in Compassion Satisfaction (R^2^ = 0.674) and 60.1% of the variance in Overall Work Experience (R^2^ = 0.601), suggesting moderate to substantial explanatory power [88,89]. In addition, the Q^2^ values for Compassion Satisfaction (Q^2^ = 0.670) and Overall Work Experience (Q^2^ = 0.554) are greater than zero, indicating strong predictive relevance of the model [80,90]. The reported effect sizes (f^2^) range between 0.166 and 0.197, indicating small to moderate effects of the predictor constructs on the endogenous variables.

As presented in Table 4, the path analysis revealed statistically significant direct and indirect relationships among the study constructs, assessed using a bootstrapping procedure with 5000 resamples to ensure robust estimation [66]. The results indicate that AHSD is positively associated with CS (β = 0.215, *p* < 0.001) and OWE (β = 0.253, *p* < 0.001), supporting H1 and H2, respectively. Furthermore, CS is positively associated with OWE (β = 0.344, *p* < 0.001), supporting H3.

The mediation analysis (H4) indicates that compassion satisfaction partially mediates the relationship between AHSD and OWE (β = 0.074, *p* = 0.001), as both the direct and indirect paths are statistically significant. The bootstrapped confidence intervals for the indirect effect do not include zero, confirming the significance of the mediating effect [91]. At the same time, these findings should be interpreted as associative rather than causal because the study relies on a cross-sectional design. Accordingly, causal inferences cannot be drawn from the reported paths, and the observed relationships may also reflect alternative directional patterns. For example, dentists with higher overall work experience may be more willing to engage in after-hours work or may evaluate such demands more positively, indicating that reverse causality cannot be ruled out.

Overall, the findings suggest that AHSD is associated with OWE both directly and indirectly through compassion satisfaction, reflecting a dual pathway in which work demands are linked to professional well-being outcomes depending on the availability of psychological resources. However, the temporal direction of these relationships should be interpreted with caution and confirmed through longitudinal research.

### 4.3. Moderation and Moderated Mediation Analysis

**Figure 3:**
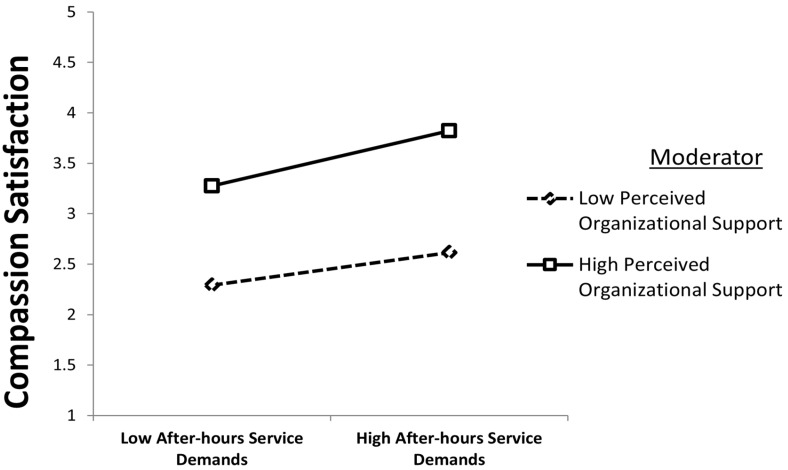
Moderating effect of POS on the AHSD–CS relationship.

The final phase of the analysis examined the moderating role of POS on the relationships among the key study variables. As shown in Table 5, POS was positively associated with the strength of the relationship between AHSD and CS (β = 0.054, *p* < 0.010), supporting H5. Although statistically significant, the magnitude of this moderating effect is relatively small (f^2^ = 0.041), which falls within the small-effect range according to Cohen’s [89] guidelines. This indicates that POS does not exert a dominant influence but provides an incremental contribution in shaping how dentists interpret after-hours demands. The simple slope analysis (Figure 3) indicates that the association between AHSD and CS is stronger at higher levels of POS, suggesting that supportive organizational contexts may modestly facilitate more favorable interpretations of after-hours demands.

**Figure 4:**
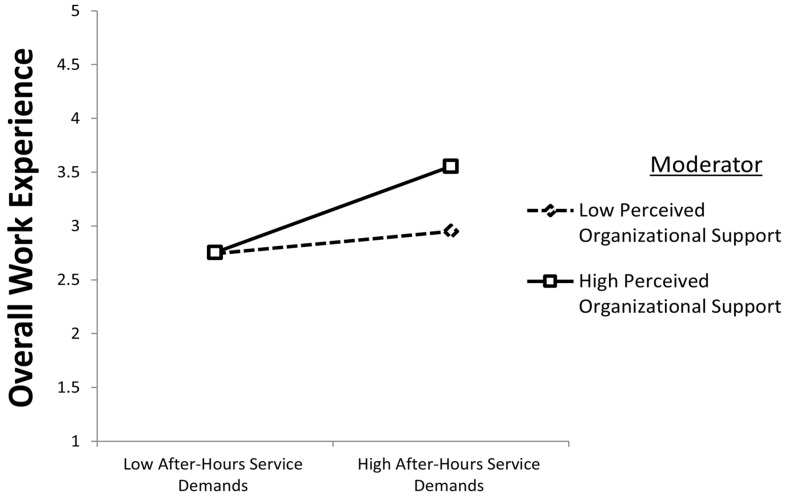
Moderating effect of POS on the AHSD–OWE relationship.

Similarly, POS was positively associated with the relationship between AHSD and OWE (β = 0.048, *p* < 0.010), supporting H6. As illustrated in Figure 4, this interaction indicates that higher levels of POS are linked to a stronger association between AHSD and OWE. However, the relatively small coefficient suggests that POS operates as a complementary rather than dominant boundary condition. From a practical perspective, this implies that organizational support alone is unlikely to substantially transform work experiences, and its contribution should be understood as incremental rather than decisive, even when combined with other psychological resources such as compassion satisfaction.

To further examine whether POS conditions the indirect relationship between AHSD and OWE through CS, a moderated mediation analysis was conducted using Hayes’ [92] PROCESS macro (Model 14) with 5000 bootstrap samples and 95% confidence intervals [93]. The results revealed a significant moderated mediation index (0.096, 95% CI [0.016, 0.168]), supporting H7. This indicates that the indirect association between AHSD and OWE through CS varies across levels of POS.

**Figure 5:**
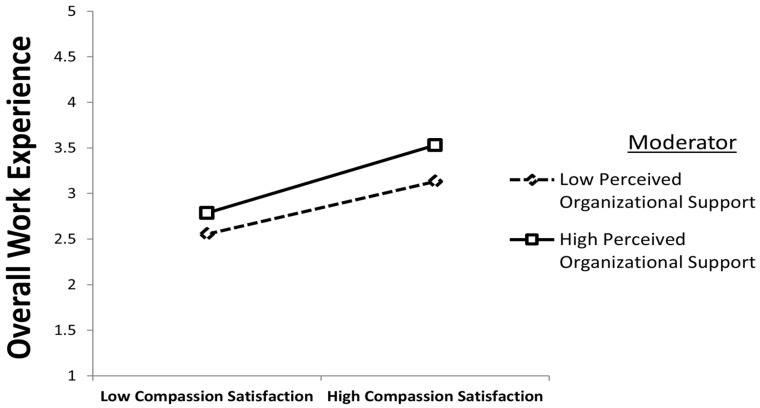
Moderated mediation effect of POS on the indirect AHSD–OWE relationship through CS.

Importantly, while this conditional indirect effect is statistically significant, its magnitude suggests a modest amplification effect rather than a substantial shift in the overall relationship. In practical terms, this finding indicates that organizational support may incrementally strengthen the role of compassion satisfaction, but its effect is modest and should not be interpreted as substantially offsetting the challenges associated with after-hours service demands. As shown in Figure 5, higher levels of POS are associated with a stronger indirect relationship between AHSD and OWE through CS; however, the effect size indicates that organizational support enhances rather than fundamentally alters the underlying mechanism.

These moderation and moderated mediation results should also be interpreted within the limits of the study’s cross-sectional design. Although POS is associated with stronger direct and indirect relationships, the analysis does not establish temporal ordering or causal direction. It remains possible, for instance, that dentists who report more favorable work experiences are also more likely to perceive greater organizational support or to engage more willingly in after-hours duties. Therefore, the reported moderation patterns indicate conditional associations rather than causal interaction effects.

Collectively, these findings demonstrate that POS functions as a contextual resource that modestly and incrementally strengthens both the direct and indirect associations between after-hours service demands and work-related outcomes, rather than acting as a primary driver of these relationships.

## 5. Discussion and Implications

### 5.1. Discussion of Key Findings

The findings provide a context-sensitive account of how after-hours service demands (AHSD), compassion satisfaction (CS), and perceived organizational support (POS) are associated with dentists’ overall work experience (OWE) in Libya. Consistent with H1, AHSD was positively associated with OWE, but this association should be interpreted cautiously and should not be read as evidence that heavier workload improves well-being. In the Libyan dental context—where workforce instability, limited institutional resources, and economic pressures are prevalent—after-hours work may be interpreted by some dentists as a professionally meaningful obligation rather than only a burden [2,62]. However, alternative explanations are equally plausible, including self-selection effects, whereby more committed or resilient dentists may disproportionately take on such work, and reverse causality, whereby dentists with more favorable work experiences may be more willing to accept after-hours duties. Accordingly, this finding is better understood as a bounded, non-causal association rather than evidence that extended work is inherently beneficial.

Supporting H2 and H3, AHSD was positively associated with CS, and CS was positively associated with OWE, suggesting that emotionally meaningful patient care may help some dentists interpret demanding work more positively, particularly where treatment outcomes are immediate and visible [20,22]. The mediation result for H4 further indicates that this association operates partly through CS, consistent with the possibility that fulfillment derived from urgent patient care contributes to more favorable overall work evaluations [94,95]. Given the cross-sectional design, however, this indirect pattern should not be interpreted causally, as dentists with higher compassion satisfaction may also be more likely to report stronger work experience and greater willingness to engage in after-hours service.

Regarding H5–H7, POS significantly moderated the AHSD–CS and AHSD–OWE relationships and strengthened the indirect path through CS. These interaction effects were small in magnitude and should be interpreted as incremental rather than substantial; supportive organizational conditions may modestly shape how demands are experienced but should not be treated as powerful standalone intervention levers [16,37]. The study also relied on self-reported, single-source data, and the pattern of fully significant relationships raises the possibility of common method inflation; although statistical checks were conducted, they reduce rather than eliminate this concern.

### 5.2. Theoretical Implications

This study contributes to the literature by offering a bounded, dentistry-specific, and Libya-contextualized theoretical interpretation of after-hours service demands. First, the findings suggest that AHSD in dentistry may not function only as a harmful demand, but may also be associated with more favorable work-related evaluations under particular contextual conditions. The theoretical relevance of this finding lies not in claiming that demanding work is beneficial, but in showing that the meaning attached to such work may matter in how it is experienced. In dentistry, where urgent care often involves immediate pain relief, visible outcomes, and direct patient appreciation, after-hours work may carry professional meaning that is less available in some other settings [5,8]. This should be understood as a context-specific and profession-specific insight rather than a generalizable claim about healthcare work broadly.

Second, the study clarifies the role of compassion satisfaction as a meaning-based psychological mechanism. The mediation results suggest that CS may help explain why demanding service obligations are sometimes associated with more favorable work evaluations by functioning as an interpretive pathway linking urgent patient care, professional fulfillment, and overall work experience [13,95]. This contribution is specific to the dental context studied here and should be interpreted cautiously within the empirical limits of a cross-sectional, self-reported design.

Third, the findings refine the interpretation of POS as a contextual resource. Rather than suggesting that organizational support fundamentally transforms demanding work conditions, the results indicate that POS may modestly and incrementally shape how such demands are interpreted [16,37]. More broadly, the study contributes a dentistry-specific perspective to demand–resource research by highlighting the distinctive combination of clinical autonomy, urgent care exposure, direct patient interaction, and limited team-based support structures as a context in which work demands, emotional meaning, and organizational support interact [6,7,96]. However, these broader theoretical observations remain suggestive and bounded by the study’s empirical scope; they should not be generalized beyond the Libyan dental context without further replication.

### 5.3. Practical Implications

The findings of this study provide cautious and context-specific implications for dental institutions, healthcare administrators, and professional bodies seeking to support the well-being of dentists. First, the results suggest that after-hours work should not be understood only as a scheduling issue, but also as an experience that may be interpreted differently depending on professional meaning and support conditions. This does not imply that after-hours demands are beneficial; rather, it suggests that how such demands are structured and recognized may matter for how they are experienced. Accordingly, dental institutions may consider structuring after-hours service more carefully through rotating emergency duty schedules, capped on-call hours, and recovery periods following extended shifts. Modest forms of financial and non-financial recognition—such as overtime compensation, emergency service allowances, formal acknowledgment, or patient appreciation mechanisms—may also help reinforce the perceived value of after-hours contributions, although these measures should be understood as incremental supports rather than solutions to the structural challenges of demanding work conditions.

Second, given the mediating role of compassion satisfaction, organizations may also consider low-intensity interventions that support dentists’ emotional resources, such as peer-support groups, brief reflective debriefing after emergency cases, and compassion-focused professional development. These measures may be particularly relevant in demanding settings where emotional exhaustion accumulates over time. Third, because the moderation effects of POS were statistically significant but small in magnitude, organizational support should be framed as an incremental rather than transformative resource. Realistic layered support may therefore be more appropriate than broad policy-level claims, and may include clearer communication about after-hours expectations, transparent workload allocation, some scheduling flexibility, access to confidential counseling, and complementary support from professional associations or dental councils. Overall, the practical value of this study lies in suggesting that relatively small, targeted support mechanisms may modestly improve how demanding work is experienced, particularly in resource-constrained settings, without implying that organizational interventions can substitute for structural reform of workload conditions.

### 5.4. Limitations and Future Research

This study has four limitations that bound its conclusions. First, the cross-sectional design prevents causal inference; all associations should be interpreted as non-directional, and reverse causality is plausible—dentists with more favorable work experiences may be more willing to accept after-hours duties, while more committed or economically motivated dentists may self-select into such work. Second, reliance on single-source self-reported data raises the possibility of common method inflation; although procedural remedies and statistical checks were applied, the pattern of fully significant hypothesized paths suggests that some associations may partly reflect shared response tendencies, and this should be treated as a substantive interpretive limitation rather than a resolved concern. Third, the removal of reverse-coded POS items (POS5 and POS7) may reflect not only a psychometric issue but also scale adaptation limitations, response-style effects, or contextual mismatch in how organizational support is understood in this setting, which may have affected measurement precision and should be addressed in future validation studies. Fourth, convenience sampling from a single national context limits generalizability; the findings are context-dependent and may not transfer to more stable or better-resourced healthcare systems. Future research should use longitudinal or multi-wave designs, incorporate multi-source data, and replicate the proposed relationships across other dental and healthcare contexts.

## 6. Conclusions

This study indicates that after-hours service demands are associated with dentists’ overall work experience both directly and indirectly through compassion satisfaction, with perceived organizational support providing a modest, incremental reinforcing context. The findings suggest that demanding work may, under specific conditions, coincide with professional meaning when emotional and organizational resources are present—a contribution that is specific to dentistry and bounded by the Libyan context from which it is drawn. The results are associative rather than causal and should not be taken as evidence that heavier workload inherently improves well-being. Sustaining dentists’ well-being requires attention not only to workload, but also to the emotional and contextual conditions through which demanding work is experienced.

## Figures and Tables

**Table 1:** Demographic Profile of Respondents.

Demographic Variable	Category	Frequency (*n* = 450)	Percentage (%)
Gender	Male	259	57.6
	Female	191	42.4
Age Group (years)	20–29	85	18.9
	30–39	187	41.6
	40–49	118	26.2
	50 and above	60	13.3
Education Level	Bachelor’s Degree (BDS)	278	61.8
	Master’s Degree	114	25.3
	Doctorate (PhD)	58	12.9
Years of Professional Experience	Less than 5 years	73	16.2
	5–10 years	176	39.1
	11–15 years	126	28.0
	More than 15 years	75	16.7
Type of Facility	Public Hospital/Health Center	161	35.8
	Specialized Dental Center	99	22.0
	Private Dental Clinic	143	31.8
	Polyclinic/Other	47	10.4
City/Region	Tripoli	139	30.9
	Benghazi	97	21.6
	Misrata	78	17.3
	Sabha	61	13.6
	Al Bayda	45	10.0
	Zawiya	18	4.0
	Derna	12	2.6
Work Status	Full-Time	383	85.1
	Part-Time	67	14.9

**Table 2:** Measurement model test.

Constructs	Items	Mean	Std. Deviation	Outer Loadings	VIF	CA	CR	AVE
After-Hours Service Demands (AHSD)						0.824	0.856	0.650
	AHSD1	4.493	0.630	0.686	1.157			
	AHSD2	4.398	0.603	0.825	1.949			
	AHSD3	4.395	0.617	0.791	1.817			
	AHSD4	4.246	0.615	0.752	1.573			
	AHSD5	4.284	0.667	0.801	1.596			
Compassion Satisfaction (CS)						0.879	0.895	0.664
	CS1	4.291	0.959	0.785	1.289			
	CS2	4.258	0.572	0.727	1.639			
	CS3	4.092	0.546	0.798	1.850			
	CS4	3.992	0.583	0.726	1.617			
	CS5	4.029	0.578	0.750	1.670			
	CS6	4.176	0.674	0.717	1.754			
	CS7	3.998	0.677	0.735	1.487			
	CS8	4.165	0.675	0.758	1.415			
	CS9	4.106	0.687	0.720	1.833			
	CS10	4.277	0.698	0.755	1.789			
Perceived Organizational Support (POS)						0.836	0.861	0.536
	POS1	4.006	0.703	0.815	1.354			
	POS2	4.067	0.747	0.791	1.349			
	POS3	4.095	0.537	0.876	1.452			
	POS4	4.429	0.574	0.721	1.190			
	POS5	2.364	1.061	-	-			
	POS6	4.115	0.531	0.744	1.205			
	POS7	2.768	1.150	-	-			
	POS8	3.232	0.996	0.785	1.436			
Overall Work Experience (OWE)						0.813	0.833	0.594
	OWE1	3.846	0.732	0.755	1.240			
	OWE2	4.168	0.556	0.746	1.729			
	OWE3	4.157	0.579	0.761	1.743			
	OWE4	3.672	0.698	0.685	1.109			
	OWE5	4.162	0.720	0.792	1.670			
	OWE6	4.146	0.650	0.723	1.733			
	OWE7	3.689	0.724	0.762	1.488			
	OWE8	3.476	0.729	0.892	1.385			

Note: Variance Inflation Factor (VIF), Cronbach Alpha (CA), Composite Reliability (CR), Average Variance Extracted (AVE). POS5 and POS7 were reverse-coded items removed due to low loadings.

**Table 3:** Discriminant validity test.

Constructs	AHSD	CS	OWE	POS
Heterotrait–Monotrait (HTMT) ratio				
AHSD	0			
CS	0.595	0		
OWE	0.610	0.641	0	
POS	0.655	0.640	0.678	0
Fornell–Larcker criterion				
AHSD	0.742			
CS	0.683	0.785		
OWE	0.674	0.681	0.728	
POS	0.669	0.719	0.671	0.680

Note: After-Hours Service Demands (AHSD), Compassion Satisfaction (CS), Overall Work Experience (OWE), Perceived Organizational Support (POS).

**Table 4:** Structural model results.

Paths	Relationships	Sample Estimate	Standard Error	T-Statistics	VIF	*f* ^2^	*CIs*		*p*-Values	Decision
							2.5%	97.5%		
Direct effect										
H1	AHSD → OWE	0.253	0.049	5.173	2.275	0.171	0.155	0.342	0.000	Supported
H2	AHSD → CS	0.215	0.048	4.484	2.133	0.166	0.122	0.311	0.000	Supported
H3	CS → OWE	0.344	0.060	5.683	2.067	0.197	0.222	0.463	0.000	Supported
Indirect effect										
H4	AHSD → CS → OWE	0.074	0.023	3.207	1.000	-	0.035	0.125	0.001	Supported

Note: After-Hours Service Demands (AHSD), Compassion Satisfaction (CS), and Overall Work Experience (OWE).

**Table 5:** Moderation and moderated mediation effect results.

Paths	Relationships	Sample Estimate	T-Statistics	*p*-Values	*CIs*		Results
					2.5%	97.5%	
Interaction effect							
H5	POS × AHSD → CS	0.054	3.007	0.003	0.014	0.087	Supported
H6	POS × AHSD → OWE	0.048	2.619	0.009	0.013	0.086	Supported
H7	Conditional indirect effect of AHSD on OWE via CS at different levels of POS	0.096	-	0.002	0.016	0.168	Supported

Note: After-Hours Service Demands (AHSD), Compassion Satisfaction (CS), Overall Work Experience (OWE), Perceived Organizational Support (POS).

## Data Availability

The data from this study can be requested from the corresponding author, Fatma Mansour Abdulmawla. The data are not publicly available due to privacy restrictions.

## Appendix A

**Table A1:** Full Measurement Scale.

Construct	Item Code	Measurement Items	Source
After-Hours Service Demands (AHSD)	AHSD1	I am frequently required to provide dental care beyond regular working hours.	Bakker and Demerouti [72]; Xanthopoulou et al. [73]
	AHSD2	Emergency dental cases often extend my working day or delay my rest time.	
	AHSD3	I am often contacted about work-related matters during my personal hours.	
	AHSD4	My workload increases significantly when performing after-hours duties.	
	AHSD5	After-hours responsibilities make it difficult for me to recover and rest properly.	
Compassion Satisfaction (CS)	CS1	I get satisfaction from being able to help people.	Stamm [74]
	CS2	I feel invigorated after working with those I help.	
	CS3	I like my work as a healthcare professional.	
	CS4	I am proud of what I can do to help others.	
	CS5	My work makes me feel satisfied.	
	CS6	I have happy thoughts about those I help and how I could help them.	
	CS7	I feel that my work makes a positive difference in others’ lives.	
	CS8	I am proud of the contribution I make to my organization.	
	CS9	I feel optimistic about my ability to help people.	
	CS10	I am pleased with how I am able to help others in my work.	
Perceived Organizational Support (POS)	POS1	My organization really cares about my well-being.	Eisenberger et al. [34]
	POS2	My organization values my contribution to its success.	
	POS3	My organization strongly considers my goals and values.	
	POS4	Help is available from my organization when I have a problem.	
	POS5	My organization shows little concern for me. (reverse-coded)	
	POS6	My organization takes pride in my accomplishments at work.	
	POS7	Even if I did the best job possible, my organization would fail to notice it. (reverse-coded)	
	POS8	My organization is willing to help me when I need a special favor.	
Overall Work Experience (OWE)	OWE1	Overall, I am satisfied with my work experience.	Zheng et al. [75]; Schaufeli et al. [76]
	OWE2	My work provides me with a sense of fulfillment.	
	OWE3	I feel proud of the work that I do.	
	OWE4	I am enthusiastic about my job.	
	OWE5	I feel emotionally engaged with my work.	
	OWE6	My work inspires me to give my best effort.	
	OWE7	My job contributes positively to my quality of life.	
	OWE8	Most days, I am enthusiastic and happy to come to work.	
